# The program for introduction of basic mathematical knowledge: the effects in six years old Mexican children

**DOI:** 10.3389/fpsyg.2023.1198675

**Published:** 2023-08-11

**Authors:** Yulia Solovieva, José Rodríguez Zavaleta, Andrea Celeste Rosete Carrillo, Luis Quintanar, Valeriya Plotnikova

**Affiliations:** ^1^Department of Educational Psychology and Pedagogy, Lomonosov Moscow State University, Moscow, Russia; ^2^Department of Psychology, Autonomous University of Puebla, Puebla, Mexico; ^3^Department of Psychology, Autonomous University of Tlaxcala, Tlaxcala, Mexico

**Keywords:** mathematical concepts, historical-cultural theory, basic mathematical knowledge, preparedness for school learning, activity theory

## Abstract

**Introduction:**

The process of teaching and learning mathematics in primary school represents an obstacle for both teachers and pupils. According to the historical-cultural conception of development and education, the way how intellectual concepts are initially introduced may radically affect a student’s success in learning. The historical-cultural conception of development, together with activity theory, may serve as the basis for creating a novel methodology for pedagogical work on mathematical concepts with pre-school and school children.

**Methods:**

The goal of the present study was to show the effects of work with an original program for the initial introduction of mathematical concepts to young school children. The program included reflexive symbolic and logical actions on the materialized and perceptual level, which were introduced and performed collectively by six-year-old children under the guidance and assistance of a teacher. The pupils were tested before and after their work with the program.

**Results:**

The results showed important qualitative and quantitative progress by the children in solving the tasks of the assessment, together with an increment of reflection on their intellectual actions.

**Discussion:**

The study points to the necessity for more intensive and extensive research, involving specialists in psychology and pedagogy trained in cultural-historical methodology.

## 1. Introduction

According to Sánchez Luján (2017), the teaching and learning of mathematics constitute a fundamental element in the field of educational sciences. Probably there is no modern society which does not consider it necessary to teach mathematics in primary school and to have an elaborated plan for study in this area (Mora Castor, 2003). At the same time, difficulties in mastering mathematics are very common and are reflected in many assessments of children’s abilities by official institutions in many countries (OCDE, 2002; OECD, 2006). The difficulties which the pupils suffer in the classroom are multiple, and cannot be explained only by developmental diseases, organic damage, or brain immaturity. The problem of the necessity of analysis of the methods of teaching, especially of initial introduction of mathematical knowledge, is a great challenge of educational and developmental psychology and pedagogy.

The difficulties of the pupils are related, among other factors, to the passive attitude of the educational authorities, as well as administrative obstacles for implementation of original methodological proposals. Some novel proposals have led to experimental studies based on the historical-cultural approach with limited groups of children and had the enthusiastic support of researchers and directors of educational programs (Zárraga et al., 2012).

Benítez and García (2011) have mentioned that mathematical competences refer to a person’s capacity to identify and understand the role of this science in the world, to make fundamental judgments, and to use this knowledge in his or her life as a thoughtful, constructive citizen. Gómez Moreno (2019) points out that the development of mathematical competences is extremely important, because it allows students to solve problems, adapt to new situations, establish relationships among diverse kinds of knowledge, and learn new concepts.

The present study was based on cultural historical conception of psychological development (Vigotsky, 1994, 1995). According to this approach, mathematical knowledge is a part of general cultural experience, acquired by the child during shared activity introduced by the adults (Talizina, 2019), which includes gradual acquisition of intellectual actions by child (Solovieva, 2022). Acquisition of intellectual actions is not a spontaneous and free process. The gradual formation of intellectual actions implies identification of the central action as an ability, which needs to be introduced and trained with the children (Galperin, 2016). The process of introduction should be planned and fulfilled by the teacher, who should organize joint solutions of intellectual problems. Such intellectual problems require of the use of numeric, logical, and symbolic means.

In this approach, the work of the teacher is organized according to the concept of the zone of proximate development, when the teacher helps the children by establishing of the questions for better orientation, demonstrating the procedures, showing examples, modeling decisions, and providing the logic of the process of each intellectual action (Vigotsky, 1995; Rosas, 2019; Solovieva, 2022; Ushakova, 2023). One of the central concepts of this psychological and pedagogical approach is the concept of orientation, which allows to design and organize the process of creation of experimental programs with their posterior approbation in groups of children.

The goal of the present study was to show the effects of an original experimental program for the initial introduction of intellectual actions with mathematical content on a group of six-year-old Mexican children, who were regular pupils of the first grade of primary school. The actions of measurement of the magnitudes of longitude, area and volume of the liquids were selected as central intellectual actions. The logical actions were related to the reflexive selection of the means for measurement and the comparison of the results. The program was designed according to the principles of cultural historical approach and psychological activity theory, which considers the psychological age of the children, the content of their intellectual actions, and the organization of constant collective interaction under the guidance of an adult. The program was based on the content of a fairy tale, so that all intellectual actions were presented as symbolic actions of the characters of the tale. The children were tested before and after their work with the program. The process of assessment was individual and included originally organized tasks (Veraksa et al., 2022).

## 2. Materials and methods

### 2.1. Participants

The participants of the study were 10 Mexican children (6 boys and 4 girls), pupils in the first grade of a private primary school in the city of Puebla. The age of the pupils was between 6, 5 and 8, 4 years old, and the average age of the children were 6, 8. The age was not essential criterion for the inclusion in the study, as it was important to include the children as regular pupils of the first grade of primary school with no previous experience of detailed work with the actions of measurement and the means of measurement. The oldest boy was included into the study because he was not able to assist the first grade of primary school due to difficult family conditions during international pandemic, so that he entered primary school 1 year later. All children were Spanish speaking pupils. The school was situated in an urban zone of medium economic level, according to the location and average economic income of the families. All the children were regular pupils who were finishing their first year of formal school education. The administration of the school agreed to take part in the study, as the school uses cultural historical methodology, based on Vigotsky’s approach. This approach considers the necessity of adult’s active orientation in the tasks of assessment, according to the conception of the zone of proximate development (Vigotsky, 1995). The study took place during the months of May and July, which represent the end of school year according to the Mexican Secretary of Education. It is important to stress that during these months the children were included into mathematic lessons, designed only according to experimental program. The children were tested before and after the work with experimental program.

The selection of the participants was based on the following criteria: (1) to be between six and 7 years old; (2) to be a regular pupil according to the norms of the educational institution; (3) to belong to the middle class of the city of Puebla; and (4) to attend most of the sessions of the experimental program as well as the sessions of initial and final assessment. The criteria of exclusion were as follows: (1) to have a history of neurological or psychiatric disturbances; and (2) to be absent during the work with the program and/or during initial or final assessment. The children’s participation in the study was voluntary and was arranged by previous agreement with the parents and the school administration.

The work with the experimental program was carried out by a teacher previously trained for work with the program content who was familiar to the children. The procedure of initial and final assessment was accomplished by two students from the Faculty of Psychology of the Autonomous University of Puebla, who were participants in the research project. The program work was done as a group, while the process of assessment was individual for each pupil before and after his work with the program. All subjects gave their informed consent for inclusion before they participated in the study. The study was conducted in accordance with the Declaration of Helsinki, and the protocol was approved by the Ethical Committee of the Department of Psychology, Lomonosov Moscow State University.

### 2.2. Design of the study

The design of the research was quasi-experimental, with the same group of participants tested before and after implementation of the experimental program. The children’s success in the solution of the problems was related to selection of appropriate external mean for the action of measure was tested before and after the work with experimental program. All tasks of the assessment and of experimental program were new for the children.

### 2.3. Instruments

Initial and final assessment were accomplished using two psychological protocols created on the basis of cultural-historical psychology, which takes into account the appropriate content for children of preschool age and the aspects of preparedness for school learning. Each participant was tested individually on selected tasks according to the protocol for the assessment of the level of preparedness for school (Quintanar and Solovieva, 2019) and on tasks from the experimental protocol for evaluation of basic mathematical knowledge (Veraksa et al., 2022).

The tasks selected from the protocol for assessment of the level of preparedness for school belonged to three spheres: (a) mediatized recollection of an object; (b) voluntary actions on verbal and graphic levels; and (c) previous mathematical abilities, such as mastery of one-to-one correspondence, seriation, and equation of conjunctions. Table 1 shows the content of the tasks selected from the protocol (Quintanar and Solovieva, 2019). It shows the sphere of the protocol and the content of each task along with the instructions and materials required for its fulfilment. The protocol was created on the basis of Vigotsky’s conception of dynamic psychological assessment, which calls for mutual collaboration between the child and an adult, and the possibility of using external help in cases of difficulties (Vigotsky, 1984; Akhutina and Pilayeva, 2008; Solovieva et al., 2021).

**Table 1:** Tasks from the protocol for assessment of the level of preparedness for school.

Sphere of development	Task	Instruction	Materials
Mediatized recall	First part of the task, at the beginning of the work with the protocol	“I shall give you this button, you must keep it for me and return it at the end of our work. When you give it to me, I shall tell you something interesting.”	The button
	Final part of the task, at the end the, the child must remember and return the button	“We have finished our work.”	The button
Voluntary sphere	Oral voluntary language	“Please, count from 10 to 1”	Oral task
	Voluntary actions in the graphic plan. *Subtask 1*	Put the pencil on the first point on the paper, draw a small straight line as I tell you, you must notice the direction of each line. All lines should be longitude, like squares in your notebook. Try not to separate the pencil from the paper and draw all the lines carefully Draw one line straight to the bottom, one line to the right, one line down, one line to the right, one line down, one line to the right. Now, continue the same sequence. What does the figure look like? What can you call this figure?	The pencil. A paper with four start points marked by an adult
	*Subtask 2*	Put the pencil on the second point. Draw a line down, one line to the right, one line up, one line to the right, one line down, one line right. Now, continue the same sequence. What does the figure look like? What can you call this figure?	The pencil. A paper with four start points marked by an adult
	*Subtask 3*	Put the pencil on the third point. Draw one line up, one line to the right, one line up, one line to the right, one line down, one line to the right, one line down, one line to the right; and now continue this sequence. What is the figure looks like? How can you call this figure?	The pencil. A paper with four start points marked by an adult
	*Subtask 4*	Put the pencil on the last point. Now, draw three lines to the right, one line up, one line to the left, one line up, three lines to the right, one line down, one line to the left, one line down, three lines to the right. What does the figure look like? What can you call this figure?	The pencil A paper with four start points marked by an adult
Previous mathematical logical abilities	Correspondence	The adult shows the child two groups of little sticks with two different colors. The quantity of sticks in two groups is almost the same and it is difficult to determine the difference visually (the difference is only one stick). The child must answer the questions as follows. Which group has more little sticks and which has less? How can we find out? If the child starts to count the sticks, the adult asks him not to do it but think about another way of discovering of the quantity of the sticks in each group. How else can we get the answer without counting? If the child does not know, the adult starts to show the action of one-by-one correspondence between the sticks of one group with the sticks of the other one. The adult asks the child to continue the action of correspondence. In cases where the child does not understand the idea, the adult completes the whole procedure	Two groups of little sticks of two different colors
	Matching of sets of sticks	After the work with the action of correspondence, the adult asks the child whether the quantity of the sticks is different in both groups. The child must show how to make the quantity equal: adding one or removing one stick to make the groups correspond (both arithmetical actions are possible)	Two groups of little sticks of two different colors
	Seriation	Copy this sequence on the paper and continue it for one line	The model of the sequence drawn on the paper (one square and two circles)

The protocol for evaluation of basic mathematical knowledge, translated from Russian into Spanish for the purposes of this study, included 17 tasks for the measurement of longitude, area, and volume (Veraksa et al., 2022). Each measurement was achieved through choosing the practical means (tools) for measuring the magnitudes. The actions of measuring included the choice of the same measure, and the understanding of the dependence of the number on the chosen measuring tool. The tasks left open the possibility of being fulfilled with the help of an adult, which shows the zone of proximate development for the practical and conceptual use of the measures as a mathematical tool.

Table 2 shows the content of the experimental protocol for evaluating the children’s basic mathematical knowledge. The table shows the sphere of the protocol and the content of each task with the instructions and materials.

**Table 2:** Tasks of the protocol for assessment of previous mathematical knowledge.

Magnitude for measure	Task	Instruction	Materials
Longitude	Task with the pencils and pens	Find the example, in which the tool was used correctly for the measurement	The model for measure
	Task with the line	Find the proper tool for measurement of the line and use it correctly	The model of the line for measurement, the pencil, the strips of paper of different sizes
	The task with horizontal strips of paper	Cut the tool correctly and choose the lines of the same size as the example	The model, the pencil, the stamen, the cutters
	The task with vertical strips of paper	Cut the tool correctly and choose lines of the same size as the example	The model, the pencil, the stamen, the cutters
	The task with the dependence between the mean and the number	Identify the number of times each tool was used to measure the same magnitude	The model, the pencil
Area	The task with carpets	Identify the examples which used the same tool correctly	The model, the pencil
	The task with the tools for measure	Identify the number of times each tool was used to measure the magnitude correctly	The model, the pencil, an example of bigger size
	The task for the measurement of an area	Choose the figures with the same tool for measurement	The model, the pencil
	Task Таngram	Choose the figures which have the same tool but a different distribution	The model, the pencil
	Task for the dependence between the number and the measure	Identify the number of times each tool was used to measure the same magnitude	The model, the pencil
The tasks for the zone of proximate development (together with an adult)	Task with the bicicle	Identify the number of the elements in each group	The model, the pencil
	Task with the flowers	Identify the number of the elements which correspond to each other in the different groups	The model, the pencil
Volume	Task with the pool	Choose the figure with the biggest volume	The model, the pencil
	Who measured correctly (1)?	Identify an example where the tool was used correctly during the measurement	The model, the pencil
	Who measured correctly (2)?	Identify the examples, where the tool was used correctly during the measurement	The model, the pencil
	Tasks by Piaget (modified)	Identify the conservation of the volume in different receptacles. Which receptacle contains more water?	The model, the pencil
	The dependence between the number and the mean chosen for measurement	Identify the number of times that each tool can be used for measuring the same magnitude	The model, the pencil

### 2.4. Procedure

The methodology of the experimental program was also based on cultural-historical psychology and activity theory applied to the process of teaching and learning. Such methodology considers psychological age of the children with the necessities of realization of interactive collective activities, which allow to guarantee the pupil’s motivation for learning of mathematics. The study was organized by academic assessors of the college who are representatives and followers of this approach in Mexico.^1^

The tasks of both protocols were presented to all participants at the beginning of the last bimester of the school year before the work with the program. The assessment was individual to each child; it took place on school premises, according to prior agreement with the director and the parents. In case of difficulties, the children received help and orientation from the adult, which did not involve completely solving the tasks. The analysis of the results considered the nature of external help used by the researcher during his work with each child. Different types of external help during the fulfilment of the protocol were also used during initial assessment.

The work with the protocol took between 45 min and 2 h. The sessions were videotaped for further analysis, according to the ethical norms and agreements with the director and the parents. The identity of the participants was never revealed.

Once the initial assessment was finalized, the experimental formative program (in writing) was presented to the participants.

The program aimed at the introduction, use, and reflection on the means of measuring longitude, area, and volumes. The tasks for the measurements were provided in different situations, which were attractive and interesting for the pupils. This was achieved by the presentation of the actions of measuring as the necessities of the characters of a fairy tale. The characters of the fairy tale found themselves in situations where the measurements necessary for achieving a goal or saving somebody were represented as a magical action. The work with the program was interactive, involving the participation of all pupils in the classroom at school and guided by the teacher. The work was programmed for planned lessons dedicated to mathematical abilities and was aimed at helping the children understand the interdependence between the means used and the magnitude in the action of measurement. The work with the program was carried out by previously trained schoolteacher known to the children. The teacher used the materials of the program prepared and explained by the researchers. The teacher did not take part in the initial and final assessment, which was fulfilled by the researchers.

The basic content of the program was related to the introduction of the actions for the measurement of longitude, area, and volume of the liquids. Different symbolic means were presented to the children to achieve the action of the measurement. All tasks were collective and interactive; so that all children took part in each task of the measurement altogether. The actions of the children were oriented and supervised by the teacher on all occasions, in line with the concept of the zone of the proximate development (Vigotsky, 1993). The basic program materials were the content of the fairy tale, the visual images of the characters, and the images of the content of the tasks needed to be fulfilled during the work with the story. Various means for measuring the magnitudes of longitude, area, and volume were used. The tools for measuring the longitudes were strips of paper of different sizes; the tools for measuring the area were paper shapes of squares and rectangles of different sizes; and the tools for measuring the volumes were glasses and other receptacles of different sizes. Table 3 presents some examples of the tasks used during the program work.

**Table 3:** Examples of the tasks used during the work with the experimental formative program for introduction of the concept of tool and measurement.

Measurement	Examples of the task
Longitude	Find and use the tools on the basis of an example; identify a figure of the same longitude
	Choose the same tools to measure different paths; determine which path is the longest and which the shortest
Area	Choose concrete tools between different choices and measure the image of the square
	Measure the area of the picture by putting different tools for measurement of the square
Volume	Serve the water in different glasses and correct the quantity of the liquid according to the standard tool
	Identify which receptacles of different sizes hold the same volume of water

A total of 16 sessions were held devoted to the work of the program. The sessions were held three times a week for 1 h. Two pupils were unable to continue the program for health reasons, so that only eight pupils finished the project.

After finishing the program, the children were assessed by the same protocols individually by the researchers. Different types of external help during the fulfilment of the protocol were also used during the final assessment.

The work with the program and final assessment were finalized in the month of July, which is the end of school year according to the official calendar of the Mexican Secretory of Public Education (SEP). Additionally, after their summer vacations, in the month of September, the children were tested in an interview conducted by the researcher. All children, who take part in the program, were included in this interview. The goal of the interview was to find out whether the children had enjoyed their work with the program, and whether they remembered its content. The session for this interview took place at the school during one session of 30 min in the presence of a new teacher assigned by the school for the second grade of primary school. The teacher who initially worked with the program did not take part in the interview, in order to exclude any influence on the children’s answers and opinions.

### 2.5. Punctuation

The children’s responses for each task of the protocols were classified and quantified by 0, 1, and 2, as shown in Table 4.

**Table 4:** Quantification of the tasks of the protocol.

Type of response	Punctuation
Correct and quick response	2
Correct and quick response after an adult’s help on the content of the task	1
Wrong response even after an adult’s help on the content of the task	0

During the fulfilment of the protocol, the children who had difficulties received help from an adult. Different types of external help were used, according to the specific content of the tasks. Table 5 shows the types of the external help provided to the children by the adult.

**Table 5:** Types of external help provided by an adult during the work with the protocol for assessment of the level of preparedness for school [9].

Task	Repeat the instruction	Show the way to find the solution (without giving the whole solution)	Detailed question or presentation of the model for the answer (without giving the whole solution)
Proposal for mediatized retention (button)	Repetition of the instruction	An example of hiding the button	Show the way to hide the button by repetition of the instruction
Recall of mediatized retention	What did I tell you when you came?	What did I give you at the beginning and what was it for?	I gave you the button and I told you to give it to me, so I would tell you something. Where is the button?
Voluntary speech	Repetition of the instruction	If we start with 10 and go backwards, which number is next?	You can see; I can count backwards as follows: 10, 9, 8. Can you continue?
Voluntary actions on graphic level 1	Repetition of the instruction	Orientation for the direction of the line on the paper	Orientation for each fragment of the line and the changes of the direction
Voluntary actions on graphic level 2	Repetition of the instruction	Orientation for the direction of the line on the paper	Orientation for each fragment of the line and changes of direction
Voluntary actions on graphic level 3	Repetition of the instruction	Orientation for the direction of the line on the paper	Orientation for each fragment of the line and changes of direction
Voluntary actions on graphic level 4	Repetition of the instruction	Orientation for the direction of the line on the paper	Orientation for each fragment of the line and changes of direction
Correspondence	Repetition of the question	Orientation by putting two pairs of sticks in two series	Orientation by showing the process of one-by-one correspondence in two series
Equalizing the groups	Repetition of the question	Orientation by showing one stick and asking: what can we do with this one?	Orientation by showing one stick and asking: we can add the stick to this group. What else can we do? (We can take one stick from another group)
Seriation	Repetition of the question	Orientation by showing the series: “look at the order of the figures, can you finish the series?”	Use of external figures (shapes) and asking the child to follow the series of external figures

Taken from Quintanar and Solovieva (2019, p. 17–41).

## 3. Results

The analysis of the data is presented below for the two protocols used for the initial and final assessments: (1) the tasks selected from the protocol for assessment of level of preparedness for school learning, and (2) those selected from the protocol for assessment of previous mathematical knowledge.

The general results obtained during the comparison of the fulfilment of the tasks selected from the protocol for assessment of level of preparedness for school learning (Quintanar and Solovieva, 2019) showed the presence of differences between the average of initial (11.250) and final (16.125) assessment (Table 6). The results were favorable for the final assessment, which pointed out that the children were able to fulfil the tasks correctly and they needed less external help from the adult.

**Table 6:** Descriptive statistics of global results obtained in the protocol for assessment of the level of preparedness for school.

Descriptive Statistics	Pretest	Posttest
Value	8	8
Absent	0	0
Mean	11.250	16.125
Typical error for Mean	0.750	1.109
Typical deviation	2.121	3.137

The student *t*-test was used for the comparison of the results of fulfilment of the tasks during initial and final assessment. This test revealed significant differences favorable to the final assessment, with a significance value of 0.001, which is significantly higher than 0.05 (*t* = −8.397, *p* < 0.001, gl = 7).

Statistical analyses of the individual results for each task showed that not all the tasks of the protocol had shown statistical differences (Table 7), but all the tasks showed better results in execution during the final assessment, compared with the initial assessment.

**Table 7:** Descriptive statistics of global results obtained for each task of the protocol for assessment of the level of preparedness for school.

	Value	Absent	Mean	Typical error for Mean	Standard deviation
1A	8	0	1.375	0.263	0.744
1B	8	0	2.000	0.000	0.000
2A	8	0	0.750	0.164	0.463
2B	8	0	1.625	0.183	0.518
3A	8	0	1.375	0.183	0.518
3B	8	0	1.625	0.183	0.518
4A	8	0	1.625	0.183	0.518
4B	8	0	1.875	0.125	0.354
5A	8	0	1.375	0.324	0.916
5B	8	0	1.750	0.250	0.707
6A	8	0	1.000	0.327	0.926
6B	8	0	1.125	0.350	0.991
7A	8	0	0.500	0.267	0.756
7B	8	0	1.250	0.313	0.886
8A	8	0	0.875	0.125	0.354
8B	8	0	1.500	0.189	0.535
9A	8	0	1.625	0.263	0.744
9B	8	0	1.875	0.125	0.354
10A	8	0	0.625	0.324	0.916
10B	8	0	1.500	0.327	0.926

A corresponds to the initial assessment.

B corresponds to the final assessment.

The tasks of correspondence and equation of groups and seriation showed significant differences (Table 8).

**Table 8:** Contrast *T* for paired samples of global results obtained for each task of the protocol for assessment of the level of preparedness for school.

Measure 1	Measure 2	*t*	gl	*p*
1A	-1B	NaN		
2A	-2B	−3.862	7	0.006
3A	-3B	−1.528	7	0.170
4A	-4B	−1.528	7	0.170
5A	-5B	−1.426	7	0.197
6A	-6B	−0.314	7	0.763
7A	-7B	−2.393	7	0.048
8A	-8B	−2.376	7	0.049
9A	-9B	−1.528	7	0.170
10A	-10B	−2.497	7	0.041

Contrast of student *t*-tests.

A corresponds to initial assessment.

B corresponds to final assessment.

ᵃThe variance of 1B is equal to 0.

The results of fulfilling the protocol for assessment of previous mathematical knowledge were compared before and after the work with experimental program. These results were analysed with the statistical program JASP, version 0.16.3.0, which evaluated two student t-tests for two conditions of the protocol: one for the global results and the other for each task carried out before and after implementation of the program with significant value of *p* < 0.05.

The comparison of the global results for the fulfilment of all the tasks of the protocol for assessment of basic mathematical abilities (in writing), found significant differences between the initial and final assessments. The median of the correct answers in the initial assessment was 14.313; and in the final assessment it was 20.875 (Table 9).

**Table 9:** Descriptive statistics of global results in initial and final assessment during fulfilment of the tasks of the protocol for assessment of previous mathematical knowledge.

Descriptive Statistics	Pretest	Posttest
Value	8	8
Absent	0	0
Median	14.313	20.875
Typical error for Median	1.785	1.302
Typical deviation	5.049	3.682

The student t-test results had a significance value of p 0.007 (*t* = −3.792, gl = 7), which was significantly lower than 0.05. This data allows to conclude that the differences between the results of initial and final assessment were statistically significant. The children’s total results were much higher in the final assessment.

The results were also analysed for each concrete task of the protocol, to establish which tasks showed differences between the initial and final assessments. Table 10 shows the data obtained by the student *t*-tests for each task.

**Table 10:** Statistical Description of the results in tasks of the protocol of evaluation of previous basic mathematical knowledge.

	Value	Absent	Medin	Typical error for Median	Standard deviation
1A	8	0	1.875	0.125	0.354
1B	8	0	1.500	0.327	0.926
2A	8	0	0.875	0.295	0.835
2B	8	0	1.500	0.189	0.535
3A	8	0	0.875	0.227	0.641
3B	8	0	1.250	0.164	0.463
4A	8	0	0.625	0.263	0.744
4B	8	0	1.000	0.189	0.535
5A	8	0	1.000	0.327	0.926
5B	8	0	1.625	0.183	0.518
6A	8	0	1.375	0.183	0.518
6B	8	0	1.375	0.183	0.518
7A	8	0	1.313	0.340	0.961
7B	8	0	1.625	0.263	0.744
8A	8	0	0.250	0.164	0.463
8B	8	0	0.750	0.164	0.463
9A	8	0	0.625	0.183	0.518
9B	8	0	0.875	0.125	0.354
10A	8	0	1.500	0.327	0.926
10B	8	0	1.875	0.125	0.354
11A	8	0	0.875	0.125	0.354
11B	8	0	1.000	0.000	0.000
12A	8	0	0.750	0.164	0.463
12B	8	0	0.875	0.125	0.354
13A	8	0	0.875	0.125	0.354
13B	8	0	0.875	0.125	0.354
14A	8	0	0.375	0.183	0.518
14B	8	0	0.875	0.125	0.354
15A	8	0	0.125	0.125	0.354
15B	8	0	0.500	0.189	0.535
16A	8	0	0.500	0.327	0.926
16B	8	0	2.000	0.000	0.000
17A	8	0	0.625	0.263	0.744
17B	8	0	1.375	0.263	0.744

A corresponds to initial assessment.

B corresponds to final assessment.

Table 11 indicates the presence of significant differences in performance between tasks 2, 8, 14, and 17. All these tasks are related to the measurement of magnitudes and showed significantly better results in the final assessment. In the rest of the tasks differences also existed, but the degree of difference was less. Tasks 11 and 16 did not show any statistical difference between the initial and final assessments.

**Table 11:** Contrast *T* for paired samples in the results in the tasks of the protocol for evaluation of previous basic mathematical knowledge.

Measure 1	Measure 2	*t*	gl	*p*
1A	-1B	NaN		
2A	-2B	−3.862	7	0.006
3A	-3B	−1.528	7	0.170
4A	-4B	−1.528	7	0.170
5A	-5B	−1.426	7	0.197
6A	-6B	−0.314	7	0.763
7A	-7B	−2.393	7	0.048
8A	-8B	−2.376	7	0.049
9A	-9B	−1.528	7	0.170
10A	-10B	−2.497	7	0.041
11A	-11A	NaN^a^		
12A	-12B	−0.552	7	0.598
13A	-13B	0.000	7	1.000
14A	-14B	−2.646	7	0.033
15A	-15B	−2.049	7	0.80
16A	-16B	NaN^b^		
17A	-17B	−2.393	7	0.048

Contrast of student *t*-tests.

A corresponds to initial assessment.

B corresponds to final assessment.

ᵃThe variance of 11B is equal to 0.

b The variance for 16B is equal to 0.

**Figure 1:**
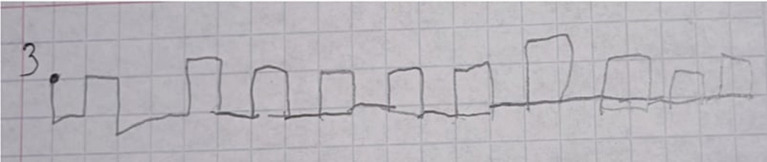
The task of graphic dictation during the initial assessment.

**Figure 2:**
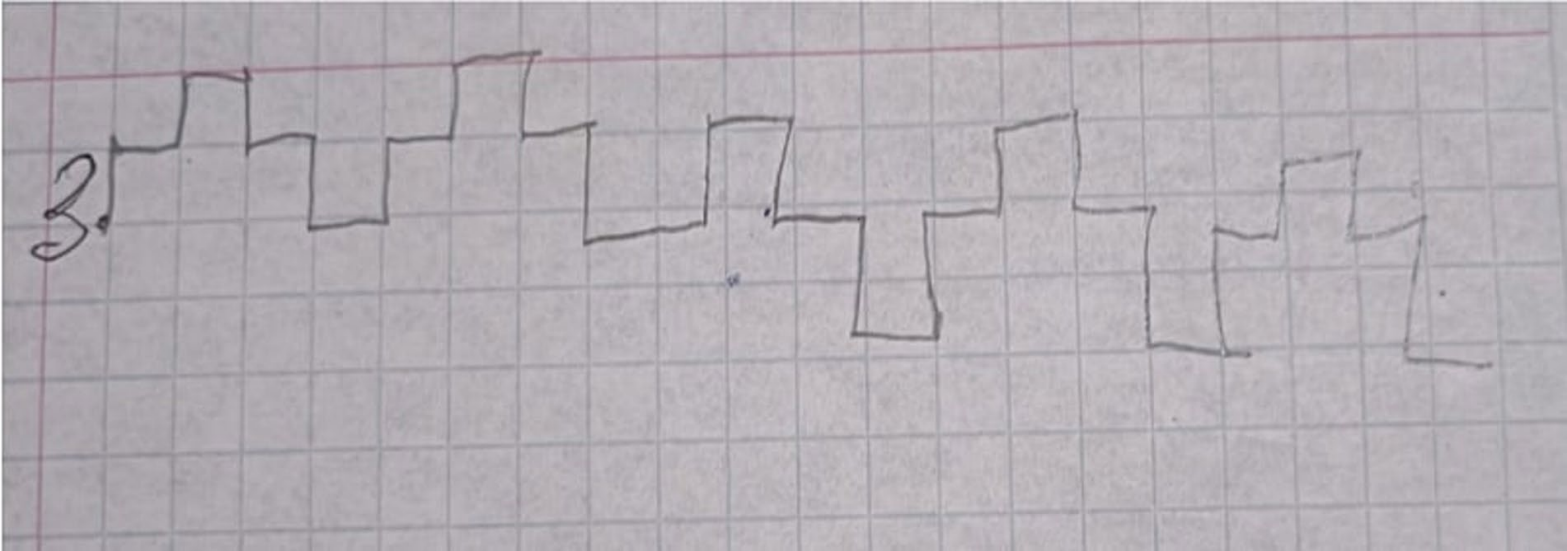
The task of graphic dictation during the final assessment.

The Figures 1, 2 show fulfilment of the task of graphic dictation from the Protocol for assessment of preparedness for school learning. In this task, the child has to follow oral instructions of an adult and draw graphic sequences on the paper.

Figure 1 shows the execution of the task of graphic dictation for the protocol of assessment of preparedness for school during the initial assessment. In this example it is possible to notice the difficulties of the child, who is not able to follow oral instructions of the adult for drawing the sequence of graphic lines.

Figure 2 shows the execution of the same task during the final assessment, in which there is no mistakes.

**Figure 3:**
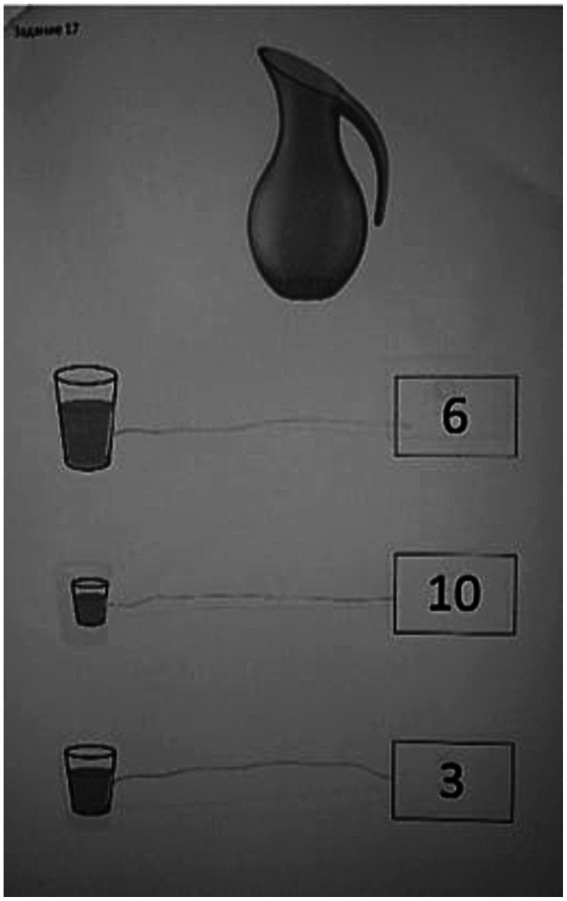
The task of identifying the correct use of the method for measuring liquid during the initial assessment.

**Figure 4:**
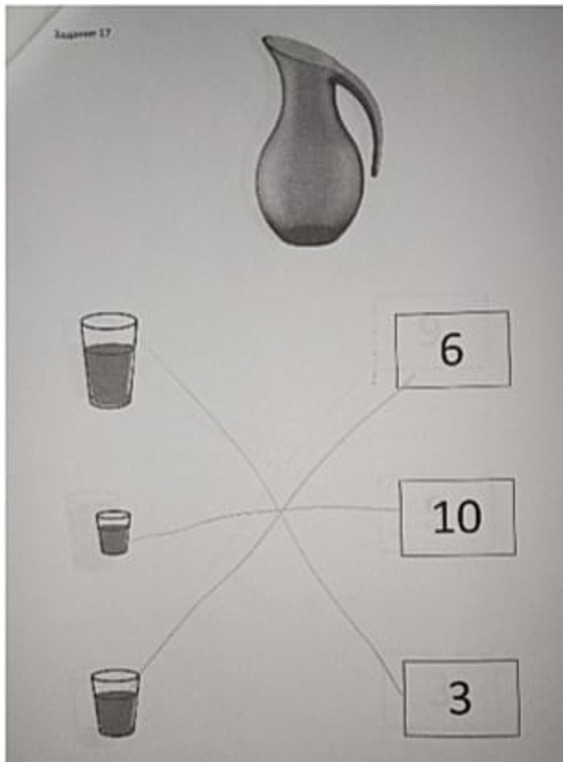
The task of identifying the correct use of the tool and method for measuring liquid during the final assessment.

The Figures 3, 4 show the examples of execution of the task from the protocol of assessment of previous mathematical knowledge. In these tasks the child has to establish the proper way for measuring the volume of the liquid.

Figure 3 shows the execution of the task of identification of correct use of the method for measuring liquid, in which the child commits a mistake by choosing wrong way for measuring. On the contrary, Figure 4 shows the correct execution of the same task during the final assessment.

### 3.1. The interview of the participants

During the interview, applied to all children after participation in experimental program, the pupils were asked questions by an adult about the content of the fairy tale and their work with the program. Below we present the collective answers of the children, who took part in the program.

Researcher: Do you remember the fairy tale, with which you worked before your summer vacations?

Participants: Yes!

Researcher: Well, we shall ask you some questions about this tale. Do you remember what it was about?

Participants: Yes, it was about how to measure.

Researcher: Yes, it was about measurement. Do you remember the main protagonist of the tale?

Participants: Yes, the prince.

Researcher: That’s true, it was the prince. Do you remember what he did?

Participants: He was helping others.

Researcher: He was traveling everywhere and helping the people. Do you remember the tasks which he had to solve?

Participants: He had to measure.

Researcher: What was the easiest measurement task?

Participants: To measure the water.

Researcher: Which tasks did you like most?

Participants: We liked the task with the cave, with the water, and with the magic sticks.

Researcher: Which were the tasks you did not like, or that you liked less?

Participants: The task with the shields.

Researcher: Why?

Participants: Because the shields were moving all the time and the wind took them away.

Researcher: Do you remember any other task which you did not like?

Participants: No, that was the only one.

Researcher: Did you like to study mathematics with the tale, or is it better without the tale?

Participants: It was better with the tale because it was fun.

Researcher: Did you like this tale?

Participants: Yes, because the tasks were very funny.

Researcher: Very well. Do you remember some tasks which we had to solve using sticks, nets, threads, and rectangles, while we were measuring different figures?

Participants: Yes.

Researcher: Was it easy or difficult when we did it for the first time?

Participants: It was easy.

Researcher: And what about the second occasion; was it easier or was more complicated?

Participants: Easier.

Researcher: Do you believe that the fairy tale helped you to answer?

Participants: Yes.

Researcher: Do you remember what was to be measured in these tasks?

Participants: Some sticks.

Researcher: Were these tasks easy?

Participants: Yes, easy.

Researcher: Well, thank you very much.

## 4. Discussion

The analysis of the results of the study shows that the children performed better during the final assessment, after their participation in the experimental program. Some results of the execution of the tasks were significantly different, and favorable during the final assessment. For example, in the protocol of assessment of the level of preparedness for school, the tasks of correspondence and equation of groups, and seriation showed significant differences, as the children were able to fulfil these tasks correctly without any help from the adults.

The examples of the executions of the tasks during the initial and final assessments showed important improvement. The final assessment showed correct fulfilment of the proposed tasks and no mistakes. Most of the children were able to fulfil the tasks correctly during the final assessment, while it was not possible during initial assessment.

It is necessary to stress that the tasks on the assessment were not included in the content of the program, which was directed to providing tasks of reflective and conscious actions of measurement of different magnitudes in the context of the fairy tale. The results of the research point out that the program helped the children improve their ability to solve logical tasks which are essential for the initial introduction of mathematical concepts. The children’s participation in the final interactive interview showed that they remembered the content of the program, and the actions and means (tools) used for measurement, and that they enjoyed working with most of the proposed tasks. They were also able to detect the most difficult and confusing tasks of the program. This data shows positive effects of collective organization of children’s work with the use of mathematical intellectual actions. Such work guarantied positive emotions and attitude toward mathematical knowledge in children. This data supports the idea of the previous publication about positive effects of the work of young scholar in teams (Glozman and Plotnikova, 2021; Sidneva et al., 2022). The study allows to think that introduction of mathematical knowledge might be presented as attractive and pleasant collective activity.

Piaget (Piaget, 1954), in his studies, discovered the importance of elementary logical actions for further acquisition of mathematical abilities in school. At the same time, the followers of Piaget, who represent the constructivist approach to teaching mathematics, did not propose any kind of training to develop logical actions. Rather, the constructivist approach claims that logical thinking is predetermined to appear by the age of 12, so that school learning can be a “free” and “spontaneous” process. Proponents of constructivism are directed to stimulate individual solutions for tasks proposed by the teacher (SEP, 2017) or provide guidance by physical sensory models of groupings and unions (Montessori, 2004; Navarro and Larrea, 2018).

On the contrary, representatives of the cultural-historical approach and activity theory in psychology claim that logical actions do not appear spontaneously but require specially organized work with the children at school (Talizina, 2018, 2019). In this sense, it’s possible to affirm that mathematical knowledge is not spontaneous, is not constructed individually by each child and do not appear just as the consequence of brain maturation. Mathematical knowledge is an example of cultural historical cultural experience with its specific symbolic language (Antonsen, 2021), which the child may or may not acquire during his or her life (Bell, 2021). This positive and successful acquisition depends on the methodology of teaching and guidance provided by the teacher (Rosas and Solovieva, 2019). This kind of effort to provide innovative experimental programs requires more research and creativity (Van Oers and Pompert, 2021). It is especially important in the region of Latin America, where successful learning of mathematics is rare (Sánchez and Escotto, 2013).

Unfortunately, official programs for teaching mathematics are mostly based on the constructivist way of thinking, which understands the acquisition of logical and mathematical abilities to evolve spontaneous spontaneously (Piaget, 1972). The central topic of constructivism is how to stimulate development; such a proposition does not involve tackling the problem of finding new methods of teaching (Inhelder and Cellérier, 1996). But the cultural-historical approach and activity theory applied to teaching and learning processes works on how to guarantee the positive development of logical action in most school children (Talizina, 2019).

Probably, now when international pandemic and impossibility of children having face-to-face studies have ended, it is a good time to start thinking about coherent changes in teaching and learning methodology. Such changes should aim to establish emotional and positive communication between the pupils and the teacher during classes, not expect pupils to “construct” logical knowledge independently, as constructivism claims (Díaz and Hernández, 2002). The teachers must organize the lessons and present mathematical data in attractive and understandable manner, so that the children might be involved in this sphere of knowledge emotionally and intellectually. The big challenge of the system of education is that the latest programs for teaching mathematics according to competences and active constructivism have not achieved any kind of methodologically clear solution. Particularly in Mexico, there is no clear systematic elaboration of proposals for work at the pre-school and school level for the initial introduction of mathematical concepts. International organizations have recommended reconsidering how to develop mathematical knowledge, and strengthen its functional aspect and reflexive, variable, and flexible use in multiple situations (OECD, 2006). While little things have been done in this direction, stagnation has dominated the basic methodology of teaching mathematics.

According to Carrillo et al. (2012), mathematical competence represents the capacity for using mathematics for judgment in a variety of situations; this applies even in extra-mathematical contexts, due to the broadness of possible application of mathematical actions. In our opinion, this view is completely accurate for the general aspects of mathematical concepts. At the same time, it is false, because it tends to lose sight of the specific character of mathematical knowledge in comparison with general intellectual knowledge. Specifically, such a broad interpretation of mathematical knowledge might be very useful at advanced levels of study, but not at the time of its introduction in primary school, because it does not offer any specific method for introducing mathematical concepts, which are represented by specific actions and specific concepts.

Radical changes in the method of teaching mathematics should be related to the content of methodological work in the classroom. The data of the research allows to stress that such changes are possible, based on psychological activity theory.

Psychological activity theory, developed by Leontiev (2009), offers a dynamic and dialectical vision of the process of teaching and learning. This process should be understood as complex cultural activity, which has its own goals, structure, content, and participants (Solovieva, 2019). The role of the teacher is fundamental during the teaching, especially at the initial stage of introduction of the concepts. At the same time, the teacher must be specifically prepared and able to systematize and organize the knowledge to guarantee its positive acquisition by the pupils.

According to Vigotsky (1993), such teaching leads to the psychological development of the child or, expressing the same idea in other words, “there is no good learning without previously organized good teaching.” This means that the teacher must possess a specific methodology to be used in the classroom. Frequently, the specific methodology must be created and applied in experimental groups, before being understood and used as a practical instrument in the classroom. Such is the case in the initial introduction of mathematical knowledge, which is expressed in the reflective fulfilment of symbolic and logical actions by children, which are normally poorly formed at pre-school age (Nisskaya, 2018; Talizina, 2018; González, 2021; Veraksa et al., 2022). The sequence and the content of these actions should be articulated with the help of concrete methods or programs and applied in groups of children who are starting to learn mathematics formally. In this research, specific mathematical abilities were introduced and formed in children, such as the measuring of different magnitudes. After the work with experimental program, the children were able to follow oral instructions of the adult for realization of graphic tasks, to understand the content of action of measuring of longitude, area, and volume of liquids and to choose proper means for measurement. The children were able to identify the actions of measurement fulfilled with and without mistakes and to correct these mistakes. Such clear understanding did not exist before participation in the experimental program, even though the children studied in the school which uses cultural historical methodological approach. Experimental program helped the pupils to identify properly the means for the measurement of the length, area, and volume and to understand the logical process of each measurement, which was not possible before the work with the program, according to initial and final testing.

The results of our study allow to offer a new original way for introducing innovative programs for interaction with the children in the classroom together with the teacher, which helps them achieve better results in understanding logical actions and the mutual dependence of the processes of measuring, the magnitude to be measured, and the tools which should be selected for these actions. All children, who took part in the program, were able to understand logical actions and enjoy the process of learning mathematical concepts in primary school. The results of the study might be used for improvement of the process of initial introduction of mathematical abilities. The study opens an opportunity for creative implementation of the concepts of zone of proximate development and orientation as main concepts of cultural historical approach.

One of limitations of this study was the small sample of the children who took part in the experimental program, because the private school chosen for the study works with small groups of children. Another limitation was the absence of the control group, as it was impossible to get permissions for assessment of children from other private primary school with similar characteristics. An essential advantage of the study consists in the effort of creation and implementation of new original program for introduction of initial mathematical knowledge by working with measurement as joint intellectual actions guided by the teacher, according to the conception of the zone of proximate development. The program should be taken into account as new contribution for initial introduction of mathematical actions within cultural historical approach. Our future efforts will include preparation for publication of the whole content of the experimental program in Spanish for its generalized use by teachers in primary schools and for correction of difficulties in the process of acquisition of mathematical knowledge.

## 5. Conclusion

The results of the study show significant differences between the initial and final assessment of the ability of children in the first grade of primary school to use the proper tools for the action of measurement. Such findings allow to confirm that the program used for our work on the intellectual actions of measurement in the context of a fairy tale was useful for the children’s understanding of the dependence of determining a magnitude on the tools and methods chosen for the measuring it. The pupils, who took part in the program showed interest and pleasure during the work with experimental program. Such data is relevant for design and approbation of original programs for introduction of basic mathematical knowledge. The children of form the first grade of primary school worked eagerly with the content of intellectual actions, guided by the teacher. The content of a fairy tale might be used as a pedagogical strategy, while the actual actions of measurement should be included and carried out by the children according to the content of the tale. The teaching of initial mathematical concepts should include the use of different and flexible symbolic means as tools of the children’s for introducing reflection during solution of logical problems. The experience of the work with the content of fairy tale allows to confirm that symbolic means and logic actions must not be only presented or explained but fulfilled by the children in the classroom under the guidance of the teacher. The program, used in the study, might be reproduced in different educational institutions and social contexts, where the work on introduction of initial mathematical knowledge is needed.

## Data availability statement

The original contributions presented in the study are included in the article/supplementary material, further inquiries can be directed to the corresponding author.

## Ethics statement

The studies involving human participants were reviewed and approved by the Ethical Committee of the Department of Psychology, Lomonosov Moscow State University. Written informed consent to participate in this study was provided by the participants’ legal guardian/next of kin.

## Author contributions

YS: conceptualization and supervision. YS and LQ: methodology, investigation, writing – review, and editing. JZ: software and visualization. JZ and AC: formal analysis. YS, JZ, AC, LQ, and VP: writing – original draft preparation. LQ: project administration. YS and VP: funding acquisition. All authors contributed to the article and approved the submitted version.

## Funding

This research was funded by the Russian Science Foundation, grant number 21–18-00584.

## Conflict of interest

The authors declare that the research was conducted in the absence of any commercial or financial relationships that could be construed as a potential conflict of interest.

## Publisher’s note

All claims expressed in this article are solely those of the authors and do not necessarily represent those of their affiliated organizations, or those of the publisher, the editors and the reviewers. Any product that may be evaluated in this article, or claim that may be made by its manufacturer, is not guaranteed or endorsed by the publisher.

## Footnotes

^1^ www.colegiokepler.edu.mx
