# Corrigendum: The program for introduction of basic mathematical knowledge: the effects in six years old Mexican children

**DOI:** 10.3389/fpsyg.2023.1277924

**Published:** 2023-08-29

**Authors:** Yulia Solovieva, José Rodríguez Zavaleta, Andrea Celeste Rosete Carrillo, Luis Quintanar, Valeriya Plotnikova

**Affiliations:** ^1^Department of Educational Psychology and Pedagogy, Lomonosov Moscow State University, Moscow, Russia; ^2^Department of Psychology, Autonomous University of Puebla, Puebla, Mexico; ^3^Department of Psychology, Autonomous University of Tlaxcala, Tlaxcala, Mexico

**Keywords:** mathematical concepts, historical-cultural theory, basic mathematical knowledge, preparedness for school learning, activity theory

In the published article Ushakova (2023) was mistakenly not cited in the article. The citation has now been inserted in **Introduction**, Paragraph 5, which should read:

“In this approach, the work of the teacher is organized according to the concept of the zone of proximate development, when the teacher helps the children by establishing of the questions for better orientation, demonstrating the procedures, showing examples, modeling decisions, and providing the logic of the process of each intellectual action (Vigotsky, 1995; Rosas, 2019; Solovieva, 2022; Ushakova, 2023). One of the central concepts of this psychological and pedagogical approach is the concept of orientation, which allows to design and organize the process of creation of experimental programs with their posterior approbation in groups of children.”

In the published article Sidneva et al. (2022) was mistakenly not cited in the article. The citation has now been inserted in **Discussion**, Paragraph 3, which should read:

“It is necessary to stress that the tasks on the assessment were not included in the content of the program, which was directed to providing tasks of reflective and conscious actions of measurement of different magnitudes in the context of the fairy tale. The results of the research point out that the program helped the children improve their ability to solve logical tasks which are essential for the initial introduction of mathematical concepts. The children's participation in the final interactive interview showed that they remembered the content of the program, and the actions and means (tools) used for measurement, and that they enjoyed working with most of the proposed tasks. They were also able to detect the most difficult and confusing tasks of the program. This data shows positive effects of collective organization of children's work with the use of mathematical intellectual actions. Such work guarantied positive emotions and attitude toward mathematical knowledge in children. This data supports the idea of the previous publication about positive effects of the work of young scholar in teams (Glozman and Plotnikova, 2021, Sidneva et al., 2022). The study allows to think that introduction of mathematical knowledge might be presented as attractive and pleasant collective activity.”

In the published article Van Oers and Pompert (2021) was mistakenly not cited in the article. The citation has now been inserted in **Discussion**, Paragraph 5, which should read:

“On the contrary, representatives of the cultural-historical approach and activity theory in psychology claim that logical actions do not appear spontaneously but require specially organized work with the children at school (Talizina, 2018, 2019). In this sense, it's possible to affirm that mathematical knowledge is not spontaneous, is not constructed individually by each child and do not appear just as the consequence of brain maturation. Mathematical knowledge is an example of cultural historical cultural experience with its specific symbolic language (Antonsen, 2021), which the child may or may not acquire during his or her life (Bell, 2021). This positive and successful acquisition depends on the methodology of teaching and guidance provided by the teacher (Rosas and Solovieva, 2019). This kind of effort to provide innovative experimental programs requires more research and creativity (Van Oers and Pompert, 2021). It is especially important in the region of Latin America, where successful learning of mathematics is rare (Sánchez and Escotto, 2013).”

In the published article Veraksa et al. (2022) was mistakenly not cited in the article. The citation has now been inserted in **Discussion**, Paragraph 11, which should read:

“According to Vigotsky (1993), such teaching leads to the psychological development of the child or, expressing the same idea in other words, “there is no good learning without previously organized good teaching.” This means that the teacher must possess a specific methodology to be used in the classroom. Frequently, the specific methodology must be created and applied in experimental groups, before being understood and used as a practical instrument in the classroom. Such is the case in the initial introduction of mathematical knowledge, which is expressed in the reflective fulfilment of symbolic and logical actions by children, which are normally poorly formed at pre-school age (Nisskaya, 2018; Talizina, 2018; González, 2021, Veraksa et al., 2022). The sequence and the content of these actions should be articulated with the help of concrete methods or programs and applied in groups of children who are starting to learn mathematics formally. In this research, specific mathematical abilities were introduced and formed in children, such as the measuring of different magnitudes. After the work with experimental program, the children were able to follow oral instructions of the adult for realization of graphic tasks, to understand the content of action of measuring of longitude, area, and volume of liquids and to choose proper means for measurement. The children were able to identify the actions of measurement fulfilled with and without mistakes and to correct these mistakes. Such clear understanding did not exist before participation in the experimental program, even though the children studied in the school which uses cultural historical methodological approach. Experimental program helped the pupils to identify properly the means for the measurement of the length, area, and volume and to understand the logical process of each measurement, which was not possible before the work with the program, according to initial and final testing.”

The authors apologize for these errors and state that this does not change the scientific conclusions of the article in any way. The original article has been updated.

## References

[B1] Sidneva A. N. Aslanova M. S. Bukhalenkova D. A. (2022). Features of the development of mathematical skills of first-graders in different educational programs. Vestnik Moskovskogo Universiteta. Seriya 14. Psikhologiya. 3, 119–144. 10.11621/vsp.2022.03.07

[B2] Ushakova O. S. (2023). Formation of the national system of preschool education. Mod. Preschool Educ. 1, 26–34. 10.24412/2782-4519-2023-1115-26-34

[B3] Van Oers B. Pompert B. (2021). Assisting teachers in curriculum innovation: an international comparative study. New Ideas Child Educ. Psychol. 1, 43–76. 10.11621/nicep.2021.010327409075

[B4] Veraksa N. E. Almazova O. V. Tarasova K. S. (2022). Dialectical and formal-logical thinking in senior preschoolers. Russian Psychol. J. 19, 129–149. 10.21702/rpj.2022.2.10

